# Exercise Participation during the COVID-19 Pandemic: Anxiety, Stress, and Precautionary Behavior

**DOI:** 10.3390/bs12110437

**Published:** 2022-11-09

**Authors:** Heetae Cho, Sunghoon Kim, Weisheng Chiu

**Affiliations:** 1 Department of Sport Science, Sungkyunkwan University, Suwon 16419, Korea; 2 Department of Physical Education and Sports Science, Nanyang Technological University, Singapore 637616, Singapore; 3 Department of Physical Education, Yonsei University, Seoul 03722, Korea; 4 Lee Shau Kee School of Business and Administration, Hong Kong Metropolitan University, Kowloon, Hong Kong, China

**Keywords:** exercise participation, anxiety, stress, precautionary behavior, COVID-19

## Abstract

Understanding emotion is critical, as it influences behavioral responses. In particular, anxiety is one of the most significant factors affecting individuals’ behavior during a pandemic situation. However, the effect of coronavirus anxiety on exercise behaviors has not been extensively explored in the extant literature. Therefore, this study examined the relationships among coronavirus anxiety, stress, precautionary behavior, and exercise participation. A total of 307 responses were collected from individuals who experienced the full length of the circuit breaker in Singapore. Data were analyzed using partial least squares structural equation modeling (PLS-SEM). Results showed that coronavirus anxiety had positive effects on stress and precautionary behavior. In addition, precautionary behavior played a mediating role in the relationships among coronavirus anxiety, stress, and exercise participation. The findings of this study identify how COVID-19 affected exercise participation during that period and suggest strategies to promote exercise participation, which would benefit individuals and governments.

## 1. Introduction

Within just a few months, COVID-19 impacted lives and society worldwide for the foreseeable future. In March 2020, the World Health Organization (WHO) publicly declared the coronavirus outbreak a pandemic [1], and many countries closed their borders and encouraged social distancing to prevent spread of the virus [1]. However, mortality rates worldwide surged exponentially since the outbreak of the coronavirus in December 2019 [2]. While containment of the virus has been the utmost priority of government officials and policymakers, the perceived risk of COVID-19 has changed and influenced countless lives. For example, schools were forced to close, smaller businesses struggled to stay afloat, employees were at risk of losing their jobs, and general sentiments toward the virus remained negative [3]. Such stresses compromise individuals’ exercise participation and health. Previous studies found that exercise was important to individual health and well-being and could act as a coping mechanism to alleviate stress through the production of adrenaline and cortisol, as well as the stimulation of endorphin production [4]. However, due to various obstacles during the COVID-19 pandemic, people could not participate in exercise as usual [5,6].

To explain this phenomenon, it is vital to understand the role of emotions in individual behavior [7,8]. Particularly, among diverse emotions influencing behavior during COVID-19 [9,10], anxiety has been highlighted by researchers [11,12,13]. For instance, previous studies on epidemics and pandemics show that anxiety, or the lack thereof, is an essential driver of behavior [11]. On the other hand, people with excessive anxiety are more likely to engage in socially disruptive behaviors, such as panic buying and unnecessarily surges in admissions to hospitals and clinics when they misinterpret their minor ailments as signs of serious infection [12,13]. That is, in a pandemic situation, anxiety is a critical factor influencing individuals’ behaviors.

Nevertheless, despite its importance, the role of anxiety and its effect on exercise participation have not received much attention in the fields of sport and physical activity during the COVID-19 pandemic. Previous studies examined the effects of exercise and physical activity on individual anxiety level prior to the COVID-19 pandemic [14,15,16] and during the pandemic (e.g., [17,18,19]). However, it has not been clearly investigated how anxiety affected exercise behavior during the COVID-19 pandemic. Therefore, to fill this gap in the literature, this study examined the effect of coronavirus anxiety on actual exercise participation based on the appraisal theory of emotion [7,8]. The research model comprised two mediating variables of stress and precautionary behavior to explain individual cognitive-emotional processing. The findings of this study contribute to a better understanding of how anxiety generated by COVID-19 affected exercise participation during this period and suggest strategies to increase exercise participation, which would benefit individuals and governments.

## 2. Literature Review and Hypothesis Development

### 2.1. Appraisal Theory of Emotion

The appraisal theory of emotion was first developed by Arnold [20] and later expanded by Lazarus [7,8]. Lazarus [7,8] proposed that the subjective experience of emotions is determined not only by one’s actions—as initially proposed by Arnold [20]–but also by one’s body feedback and cognitive appraisals. Broadly speaking, the appraisal theory of emotion asserts that emotions are caused and differentiated by individual appraisal of a stimulus [21]. Lazarus [7] asserts two levels of stimulus appraisal: primary and secondary appraisals. Primary appraisals refer to assessments of situations as either emotionally relevant or irrelevant. Emotionally irrelevant situations are those that do not affect one’s well-being and safety and do not require any behavioral responses (e.g., watching television at home), while emotionally relevant situations are those that can affect well-being and require behavioral intervention (e.g., suddenly hearing a loud noise outside the house and going out to find what caused it). Emotionally relevant situations are further appraised as either challenging or threatening. Challenging situations involve a potential for gain or growth, while threatening situations may lead to harm, loss, and negative consequences [7]. According to Lazarus [7], primary appraisals alone do not fully determine one’s emotions; they interact with secondary appraisals to determine emotions. Secondary appraisals refer to assessments of the availability and efficacy of the individual’s coping resources for those situations, resulting in perception of the situation as either low threat or high threat [7].

Based on the appraisal theory of emotion [7], it can be assumed that one’s appraisal of the COVID-19 situation might affect one’s feelings of anxiety. These emotions might then affect other cognitive and emotional components, such as physiological state. Furthermore, the appraisal theory of emotion states that such factors can interact with one another [21]. In other words, during the COVID-19 situation, an individual’s stress can affect his or her behavioral responses, such as taking precautionary measures, and such behaviors can further affect other behaviors (e.g., sports participation). As such, based on the appraisal theory of emotion [7,8], this study investigates how individuals’ cognitive-emotional processing affects behavioral responses during the COVID-19 situation.

### 2.2. COVID-19, Anxiety, and Stress

The COVID-19 pandemic can be perceived as a highly threatening situation, and it has been strongly correlated with numerous negative emotions and psychological outcomes for people around the world [22,23]. For instance, during the COVID-19 pandemic, individuals felt confused, shocked, and fearful [24], and quarantine lead some people to feel depressed [25]. Researchers also found that the COVID-19 pandemic was highly correlated with negative emotions, including anxiety [22,23]. Anxiety refers to an emotion that individuals experience when faced with danger and threat [26], which can result from numerous negative events, such as severe illness [27] and unemployment [28]. Furthermore, individuals also feel anxious toward a pandemic [29].

According to Adwas et al. [26], anxiety is closely related to stress, a state in which an individual perceives a threat and feels that their coping resources are inadequate [30]. It is an emergency state in which the individual is mobilized—via physiological, cognitive, and behavioral responses—to face a threat [31], indicating that anticipation or perception of a threat leads to both stress and anxiety. Daviu et al. [32] noted that not only do stress and anxiety often co-occur, but the neural areas responsible for the two are intertwined. Recently, Levkovich and Shinan-Altman [24] also found that people often felt both anxious and stressed during the COVID-19 pandemic.

Anxiety is a resultant emotion of the perceived threat, whereas stress is a physiological state that accompanies that resultant emotion of anxiety [32]. This means that anxiety often leads to stress, and this association is non-trivial and strongly rooted in individual biology. In other words, this association between stress and anxiety is universal [32]. Thus, based on previous studies, we propose the following hypothesis:

**H1.**  *Coronavirus anxiety has a positive effect on stress.*

### 2.3. Precautionary Behavior

To reduce both the spread of the COVID-19 virus and its strain on the health care systems, many countries have imposed non-pharmaceutical interventions (NPIs) [33]. NPIs refer to public health measures designed to slow the spread of a virus [34]. Examples include measures, such as social distancing, quarantine, hygiene practices, or restriction on certain activities [33]. According to Seale et al. [35], the adherence to NPIs is affected by anxiety about the infectious virus. Leung et al. [36] also found that, during the SARS outbreak in Hong Kong, individuals who were more anxious about the SARS virus were also more likely to adopt comprehensive precautionary measures or NPIs, including covering their mouths with a mask or a cloth. Conversely, the lack of an appropriate level of anxiety has been shown to lead to slower adoption rates of NPIs [37]. This indicates that anxiety about an infectious virus helps to increase adherence to NPIs by leveraging on the individual’s fear of contracting the virus; increase in one’s anxiety about COVID-19 can lead to increase in adherence or adoption of NPIs. Thus, based on previous research, this study proposes the following hypothesis:

**H2.**  *Coronavirus anxiety has a positive effect on precautionary behavior.*

Similar to the effect of anxiety on NPIs, stress has also been found to be positively associated with NPIs [38]. For instance, Novotny et al. [38] found a significant positive relationship between one’s level of stress and one’s adherence to restrictive NPIs, such as stay-home measures and wearing of masks. Furthermore, Charoensukmongkol and Phungsoonthorn [39] noted that the stress resulting from emotional strains and the perceived uncertainties during the COVID-19 pandemic led to higher adoption rates of certain NPIs within work organizations. This stress-reducing side effect may explain why as one’s levels of stress increases, one’s adherence to NPIs also increases; it may be a behavioral reaction to reduce one’s stress levels [35]. Given that stress tends to increase one’s adoption of NPIs, we suggest the following hypothesis:

**H3.**  *Stress has a positive effect on precautionary behavior.*

### 2.4. Exercise Participation during the COVID-19 Situation

Participating in sport activity has been shown to be very important in maintaining our physical [40] and psychological health [41], both of which are threatened by this COVID-19 pandemic [42]. However, due to many health measures and restrictions applied to sport and fitness facilities, it remained a challenge to engage in sports activities. To reduce both the spread of the COVID-19 virus and its strain on the health care systems, many countries have imposed public health measures, such as social distancing and complete lockdowns of the general population [33]. For example, the Singapore government implemented a similar set of public health measures, such as restricting group sports activity and closing sports facilities [43], which increased difficulty in engaging in sports activities, leading to a reduction in the levels of participation.

Previous studies have shown that negative affectivity, such as anxiety, is inversely related to sports behavior [4]. Goodwin [44] asserted that individuals with anxiety were less likely to participate in sports due to lower energy levels or apathy. Similarly, Radloff [45] found that anxious individuals had lower levels of motivation and were thus less likely to participate in sports behavior. That indicates that anxiety has a negative effect on sports participation. Similar to the relationship between anxiety and sport participation, stress was associated with negative health behaviors, including poor dietary practices and a lack of exercise [46]. Many previous studies also showed stress to negatively correlate with sports participation (e.g., [47,48]). That is, as one’s stress increases, he or she would be less likely to participate in sports or physical activities. Zillmann and Bryant [46] asserted that stress induced individuals to engage in negative health behaviors as a means of emotion-focused coping; it is emotionally easier to not exercise than it is to exercise. Thus, based on previous studies, we propose the following hypotheses:

**H4.**  *Coronavirus anxiety has a negative effect on exercise participation.*

**H5.**  *Stress has a positive effect on exercise participation.*

**Figure 1:**
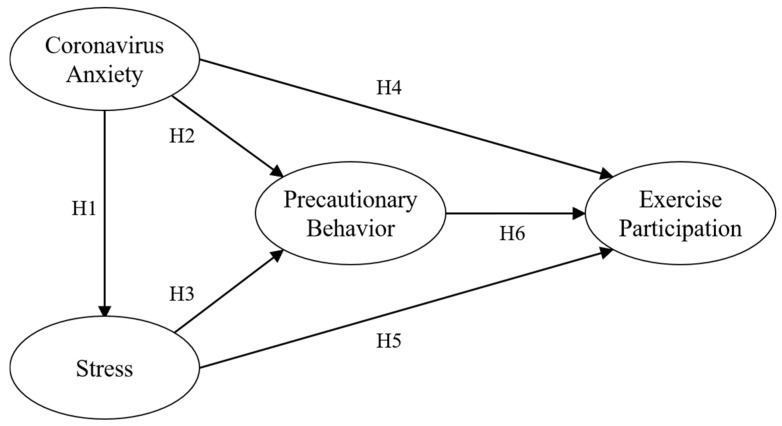
A hypothesized model.

Lastly, adhering to NPIs requires individuals to engage in behaviors that interrupt their sports participation, such as closing schools, staying at home, and prohibiting gatherings [49]. Furthermore, Lippi et al. [50] asserted that some of the most frequently performed sports activities, such as walking, cycling, and swimming, are challenging for people following NPIs. In addition, the situation can be worsened by lack of indoor physical activity as a viable option for individuals due to lack of time and equipment [50]. That is, based on the findings of previous studies, it can be assumed that adhering to NPIs interrupts and decreases sport participation. Thus, we propose the following hypothesis and research model (Figure 1):

**H6.**  *Precautionary behavior has a negative effect on exercise participation.*

## 3. Methods

### 3.1. Participants and Data Collection

During the circuit breaker period, social interaction was restricted, and strict safe distancing was implemented. Most businesses could not continue to operate during this period, except for essential services, such as banking, finance, and manufacturing [51]. In addition, all sport facilities in Singapore were closed, and limited outdoor exercise (e.g., walking, running, and cycling) was allowed when participating alone (Sport Singapore, 2020). That is, the circuit breaker measures refer to partial lockdown in Singapore.

Prior to data collection, this study’s purpose, protocols, and ethical standards were approved by the Institutional Review Board (IRB) of the first author’s university. Using the snowball sampling method, this study collected data from individuals who experienced the full length of the circuit breaker in Singapore from 7 April to 1 June 2020. The online survey was conducted through utilization of Google Forms, and this study distributed the survey through social media, including Instagram, Whatsapp, and Telegram. Prior to filling out the survey, all participants were required to read the study information and consent form. Only those who consented to participate in the research were allowed to answer the survey questions. In addition, to maintain data quality, two attention check questions were used to see if participants were paying attention when filling out the online survey. As a result, a total of 307 valid responses were collected, and the proportions of female and male participants were 54.7% (n = 168) and 45.3% (n = 139), respectively. The average age of the respondents was 23.8 years.

### 3.2. Measures

We first used one question to identify respondents’ perception of COVID-19 (i.e., how much does COVID-19 affect your life?) using an 11-point Likert scale ranging from 0 (does not affect me at all) to 10 (severely affects my life) and found that most respondents were worried about the effect of COVID-19 (M = 7.4, SD = 1.7). Next, based on the literature review, the survey instrument was developed and comprised of four sections: anxiety, stress, precautionary behavior, and exercise participation. This study used a statement to complete the survey with the recollection of respondents’ state of mind during the circuit breaker (i.e., Please note that the mindset while answering the questions should be that of the circuit breaker period of the COVID-19 pandemic from 7 April 2020, to 1 June 2020).

*Anxiety.* Individual’s anxiety during the COVID-19 circuit breaker period was measured by the Coronavirus anxiety scale developed by Lee [52]. The scale consists of 5 items, and example items are “I felt dizzy, lightheaded, or faint when I read or listened to news about the coronavirus” and “I had trouble falling or staying asleep because I was thinking about the coronavirus.” The items were rated on a 5-point, which was used to score participant’s responses from 0 (not at all) to 4 (nearly every day).

*Stress.* Individual’s level of stress during the COVID-19 circuit breaker period was assessed by Cohen et al. [53] perceived stress scale. The scale consists of 10 items, which were scored on a 5-point Likert scale from 0 (never) to 4 (very often). An example item is “During the cricket breaker, how often have you been upset because of something that happened unexpectedly?”

*Precautionary behavior.* Individual’s precautionary behavior was measured by the non-pharmaceutical Intervention scale from Lee et al. [54] and adapted for the COVID-19 situation. The scale consists of 10 items, such as “I will get information about local medical facilities in preparation for an emergency because of COVID-19 before making a trip outside of my house”; “I will frequently wash my hands while I am outside of my house”; and “I will restrain from touching my eyes, nose, and mouth while outside of my house.” A 7- point Likert scale ranging from 1 (strongly disagree) to (7 strongly agree) was used to score participants’ responses over the COVID-19 circuit breaker period.

*Exercise participation.* Respondents’ actual exercise participation was assessed by the three-item scale of Godin and Shephard [55]. The items were open-ended questions, and the items of this scale calculate the average frequency of exercise participation every week in three physical activity levels. The examples items are “how many times per week did you participate in strenuous exercise?”, “how many times per week did you participate in moderate exercise?”, and “how many times per week did you participate in mild exercise?”

### 3.3. Data Analysis

After the initial dataset was obtained, the Mahalanobis Distance examination was conducted to identify multivariate outliers [56]. As a result, 20 outliers were found and deleted, leaving 287 responses for further analysis. The common method bias was also tested using Harman’s single factor examination [57] The result revealed that the single factor was 23.73%, indicating that common method bias did not exist in this study. In addition, full collinearity assessment was used by evaluating whether the variance inflation factors (VIFs) exceed the threshold (3.3) [58]. Consequently, all VIF values were below the suggested value, supporting lack of common method bias as a concern in this study.

As a next step, partial least square structural equation modeling (PLS-SEM) was carried out using the SmartPLS software [59]. As PLS-SEM is a predictive approach, it is useful to explain and predict the study construct (i.e., exercise participation) in a complex model [60]. Moreover, PLS-SEM is a flexible approach without the requirement of normal distribution assumption, and therefore, it avoids the issue of data normality [60]. Therefore, PLS-SEM is considered an adequate tool for this study.

The minimum sample size required in this study was examined according to Hair et al.’s [60] guidelines. Because there are three predictors of an endogenous construct (i.e., physical participation level), a minimum of 103 observations was necessary to achieve a statistical power of 80% for detecting R^2^ values of 0.10 in physical participation level in the proposed model at a significance level of 5%. Therefore, the sample size of this study (n = 287) fulfills the requirements for use of PLS-SEM analysis.

## 4. Results

### 4.1. Measurement Model

The convergent validity was first evaluated by outer loadings of indicators and the average variance extracted (AVE). However, it was found that one item of stress reported low outer loadings (<0.40), and therefore, this item was removed [60]. Moreover, four additional items were deleted to produce AVE values of coronavirus anxiety and precautionary behavior higher than the suggested value of 0.50 [60]. As reported in Table 1, the AVE values surpassed the cut-off (0.50), suggesting adequate convergent validity [60]. Second, internal consistency reliability was evaluated by composite reliability (CR). The CR values ranged from 0.756 to 0.909, exceeding the recommended value (0.50) and supporting internal consistency reliability [60]. In addition, discriminant validity was examined by Heterotrait–Monotrait ratio (HTMH) [61]. As shown in Table 2, the correlations of HTMH were all lower than the conservative criterion (0.85), indicating adequate discriminant validity of measures [61].

### 4.2. Structural Model

**Figure 2:**
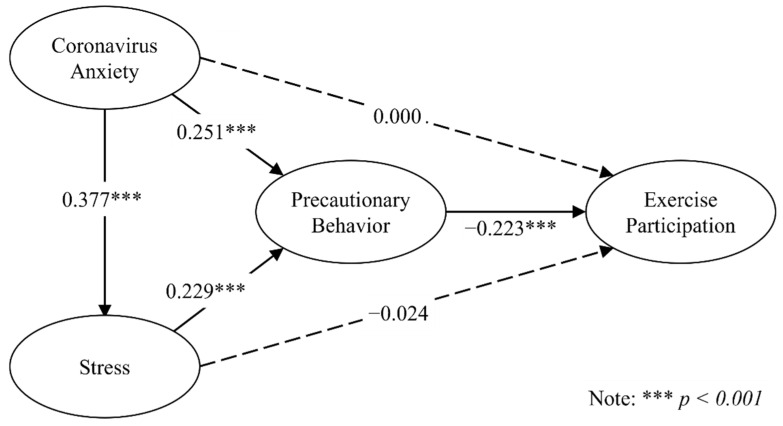
A structural model.

Hypothesized relationships in the research model were examined by a PLS bootstrapping algorithm with 2000 subsamples at a significance level of 0.05. Collinearity in the structural model was initially assessed using variance inflation factor (VIF). All VIF values were lower than 5, excluding collinearity problems in the structural model [60]. The statistics of the structural model, including path coefficient, standard deviation, and *t*-value, are reported in Table 3 and Figure 2. Coronavirus anxiety had significant and positive influence on stress (*β =* 0.377, *t* = 8.962, *p* < 0.001) and precautionary behavior (*β* = 0.251, *t* = 4.325, *p* < 0.001) but had no direct impact on exercise participation (*β* = −0.024, *t* = 0.349, *p* = 0.727). Moreover, stress had positive impact on precautionary behavior (*β* = 0.229, *t* = 3.555, *p* < 0.001) but had no direct influence on exercise participation (*β* = 0.000, *t* = 0.092, *p* = 0.997). Finally, precautionary behavior had a negative and significant influence on exercise participation (*β* = −0.223, *t* = 3.398, *p* = 0.001).

Mediation analysis was employed as in Nitzl et al. [62]. The indirect effect (*a* × *b*) in the research model was examined for significance. If a significant indirect effect was established, the second step is to decide the type of mediation by examining the significance of the direct effect (c′) [60,62]. In this study, the indirect effects were significant in the relationships between coronavirus anxiety and exercise participation (*p* = 0.047), between coronavirus anxiety and precautionary behavior (*p* = 0.002), and between stress and exercise participation (*p* = 0.027), suggesting the existence of mediations in the research model. Furthermore, analysis of specific indirect effects showed four significant indirect paths: Coronavirus anxiety → Precautionary behavior → Exercise participation (estimate = −0.056, *t*-value = 2.772, *p* = 0.007), Coronavirus anxiety → Stress → Precautionary behavior → Exercise participation (estimate = −0.019, *t*-value = 2.000, *p* = 0.046), Coronavirus anxiety → Stress → Precautionary behavior (estimate = −0.051, *t*-value = 2.211, *p* = 0.027), and Stress → Precautionary behavior → Exercise participation (estimate = 0.086, *t*-value = 3.124, *p* = 0.002) (see Table 4).

## 5. Discussion

Researchers in different academic fields, such as psychology, psychiatric disorders, and behavioral and cognitive science, have highlighted the critical impact of anxiety on individuals’ behaviors (e.g., [63]). However, the influence of anxiety on sport behavior has not been investigated. Thus, this study examined how coronavirus anxiety affected individuals’ stress, non-pharmaceutical intervention practices, and exercise participation. Based on the findings of this research, we discuss theoretical and practical implications.

### 5.1. Theoretical Implications

This study first found a significant positive effect of coronavirus anxiety on stress. This was an expected result as negative emotions are closely related to stress [64]. Similar to the finding of this study, previous studies found that, in the COVID-19 situation, increased anxiety and stress can negatively affect psychological health [65] and body image [66], resulting in increased suicide rates [67]. Furthermore, according to Shevlin et al. [68] and Tull et al. [69], the levels of anxiety and stress are increased due to COVID-19. Specifically, the perceived threat of a virus is positively associated with feelings of anxiety and the state of stress [70]. This is consistent with the aforementioned assertions that stress is an indication of a perceived threat and a stimulus to mobilize the body to respond [30]. This means that stress is an indication of the highly perceived threat of the virus, suggesting a positive relationship between perception of COVID-19 and stress through the level of anxiety an individual feel.

We also found that sport participants’ anxiety increased non-pharmaceutical intervention behavior. This suggests that the level of adherence to precautionary behavior toward a disease depends on an individual’s anxiety regarding that disease, supporting the findings of Perez-Fuentes et al. [23] about the perceived threat from COVID-19 and emotional state. Similarly, previous research has found that the perceived threat of a virus positively affected the adoption of NPIs [35]. This indicates that infection by the coronavirus would have a large emotional impact on individuals, leading to precautionary behavior.

In addition to coronavirus anxiety, we found that stress had a positive effect on non-pharmaceutical intervention behavior. As mentioned before, non-pharmaceutical intervention is a form of control meant to protect an individual from spreading a virus [71]. Such interventions include isolation of an infected patient, quarantine, border controls, and personal social distancing [72]. In the COVID-19 situation, individuals are likely to have a high level of stress, as they cannot participate in sport as before. In addition, the limited personal space in households is an essential factor, causing high stress such as that experienced by individuals in Singapore, which may result in an increase in precautionary behaviors.

Last, this study found no direct effects of anxiety and stress on exercise participation, while there were significant indirect effects of anxiety and stress on exercise participation through non-pharmaceutical intervention. The results indicate that individuals exposed to a risky situation experience emotional and cognitive processes (i.e., anxiety and stress) that influence their behavioral outcomes. The findings of this study were consistent with the appraisal theory of emotion in explaining the relationship among individuals’ cognition, emotion, and behavior [7,8]. Moreover, we found an interesting effect between behavioral responses, indicating that precautionary behavior had a negative effect on exercise participation. That is, individuals are less likely to participate in sport in the presence of precautionary behavior, although physical exercise was one of the few government-endorsed activities allowing individuals to leave home during the shutdown.

### 5.2. Practical Implications

During the circuit breaker, the Singapore government imposed numerous NPIs, such as closing sports facilities and suspending all sports activities [43], which likely reduced individuals’ levels of sports participation. In addition, due to the many health measures and restrictions applied to sports and sports facilities, it was challenging to engage in sports activities. For instance, under the ‘Advisory on tightened measures for sport and physical exercise during the extended circuit breaker period,’ all sports facilities were closed, and any exercise or sports activity had to be performed alone—with some activities also requiring use of a mask [73]. Given that there were strict NPIs on sports activities during the circuit breaker, it was difficult to find ways to continue to engage in sports activity. This increased difficulty in engaging in sports activities might have led to a reduction in sports participation. Although NPIs, such as lockdowns or stay-home measures, have been shown to reduce the spread of respiratory viruses [74], they are not sustainable for a prolonged period as they have been shown to be detrimental to physical and psychological health [75].

Previous research revealed that suicide rates have been increasing over the past few years [67]. Moreover, social isolation due to government-enforced NPIs may cause individuals with suicidal ideations to lack necessary emotional support and interaction [76]. However, it is evident that NPIs, including lockdown or quarantine measures, are effective ways to prevent virus spread, although they can produce anxiety and increase stress. This study also found a negative influence of anxiety on sports participation, which is an active coping strategy. Therefore, given the importance of exercise, we suggest that governments and public organizations need to provide individuals with opportunities to participate in exercise activities. More specifically, it is necessary to develop indoor exercise programs, and sport facilities and equipment should be distributed free of charge to maintain health. Furthermore, educational campaigns should be conducted to increase understanding and facilitate exercise motivation.

### 5.3. Limitations and Future Research

Although this study produced new insights, there are some limitations. First, we only collected data from individuals who experienced the full length of the circuit breaker in Singapore. In addition, most of the respondents were young (M = 23.8 years). Thus, future research needs to collect data from different groups of respondents in various countries to identify the generalizability of the results. The second limitation of this study is that we did not include moderating variables in the research model, such as sport participation level before the pandemic. Additionally, this study did not ask people about chronic diseases, which could significantly influence sport participation. As such, future studies should consider measuring the suggested factors to provide a comprehensive understanding of individuals’ sport participation. Lastly, coronavirus has mutated over time to show a lower fatality rate. As such, individuals may feel less anxious and more actively participate in sports compared to the early phase of the COVID-19 outbreak. Future research, thus, should consider the level of coronavirus fatality and investigate its relationship with exercise behavior. In addition, it is necessary to examine how to adapt to the new normal [77] and to identify the role of emotional contagion [78,79] to better understand individuals’ decision-making during the pandemic.

## 6. Conclusions

The coronavirus (COVID-19) has impacted individual lives and likely will continue to do so for many years. This situation has resulted in negative emotions, especially anxiety, which can change and influence behavior. As such, this study investigated how coronavirus anxiety is related to stress, precautionary behavior, and exercise participation during the pandemic situation and suggested strategies to promote exercise behavior. In addition, although the appraisal theory of emotion [7,8] has not been widely used in the field of sport, this study employed this theory to elucidate individuals’ psychological processes regarding sport participation during the COVID-19 pandemic. This study showed that the appraisal theory of emotion [7,8] is applicable in the field of sport and exercise. The findings of this study contribute to a better understanding of the effect of anxiety on exercise behavior during COVID-19 and to clarifying psychological processes during decision-making related to sports.

## Figures and Tables

**Table 1:** Psychometric properties of the measurement model.

Constructs and Items	λ
**Coronavirus anxiety** (α = 0.758, CR = 0.846, AVE = 0.579) *How often have you experienced the following activities during the circuit breaker?*	
I felt dizzy, lightheaded, or faint when I read or listened to news about the coronavirus.	0.712
I had trouble falling or staying asleep because I was thinking about the coronavirus.	0.799
I felt paralyzed or frozen when I thought about or was exposed to information about the coronavirus.	0.709
I lost interest in eating when I thought about or was exposed to information about the coronavirus.	0.735
I felt nauseous or had stomach problems when I thought about or was exposed to information about the coronavirus.	-
**Stress** (α = 0.887, CR = 0.909, AVE = 0.528) *Over the duration of the circuit breaker*	
How often have you been upset because of something that happened unexpectedly?	0.758
How often have you felt that you were unable to control the important things in your life?	0.746
How often have you felt nervous and “stressed”?	0.759
How often have you felt confident about your ability to handle your personal problems?	0.624
How often have you felt that things were going your way?	0.648
How often have you found that you could not cope with all the things that you had to do?	0.733
How often have you been able to control irritations in your life?	0.673
How often have you felt that you were on top of things? *	-
How often have you been angered because of things that were outside of your control?	0.745
How often have you felt difficulties were piling up so high that you could not overcome them?	0.829
**Precautionary behavior** (α = 0.819, CR = 0.909, AVE = 0.528)	
I will check for the information and symptoms of coronavirus (COVID-19) by visiting the website of the Ministry of Health Singapore or WTO before making a trip outside of my house.	0.849
I will read and check precautions about coronavirus (COVID-19) through doctors or general practitioners before making a trip outside of my house.	0.891
I will get the information about local medical facilities in preparation for an emergency because of coronavirus (COVID-19) before making a trip outside of my house.	0.867
I will frequently wash my hands while I am outside of my house.	-
I will restrain from touching my eyes, nose, and mouth while I am outside of my house.	-
I will keep away from those who have the symptoms of coronavirus (COVID-19) while making a trip outside of my house (Fever/ Dry Cough/ Tiredness) *	-
I will carefully keep an eye on my health condition after making a trip outside of my house.	0.607
**Exercise participation** (α = 0.543, CR = 0.752, AVE = 0.510) *Considering last month, how many times PER WEEK did you do the following kinds of exercise for more than 15 min during your free time?*	
Strenuous Exercise (Heart Beats Rapidly) (e.g., running, jogging, hockey, football, soccer, squash, basketball, judo, roller skating, vigorous swimming, vigorous long-distance bicycling)	0.868
Moderate Exercise (Not Exhausting) (e.g., fast walking, baseball, tennis, easy bicycling, volleyball, badminton, easy swimming, popular and folk dancing)	0.678
Mild/light Exercise (Minimal Effort) (e.g., yoga, archery, fishing from riverbank, bowling, horseshoes, golf, easy walking)	0.564

* Items were removed due to low indicator loadings.

**Table 2:** Discriminant validity (HTMT).

Constructs	1	2	3	4
1. Coronavirus anxiety				
2. Stress	0.450			
3. Precautionary behavior	0.415	0.327		
4. Exercise participation	0.152	0.170	0.380	

**Table 3:** Results of the structural model.

Hypotheses	Structural Paths	Standardized Coefficient (β)	Standard Deviation	*t*-Value
H1	Coronavirus anxiety → Stress	0.377	0.042	8.926 ***
H2	Coronavirus anxiety →Precautionary behavior	0.251	0.058	4.325 ***
H3	Stress → Precautionary behavior	0.229	0.064	3.555 ***
H4	Coronavirus anxiety → Exercise participation	−0.024	0.069	0.349
H5	Precautionary behavior → Exercise participation	−0.223	0.066	3.398 ***
H6	Stress → Exercise participation	0.000	0.092	0.003

Note: *** *p* < 0.001.

**Table 4:** Results of total and specific indirect effects

Path	Standardized Estimate	Standard Deviation	*t*-Value
Total indirect effects			
Coronavirus anxiety → Exercise participation	−0.075	0.038	1.992 *
Coronavirus anxiety → Precautionary behavior	0.086	0.028	3.124 **
Stress → Exercise participation	−0.051	0.023	2.211 *
Specific indirect effects			
Coronavirus anxiety → Precautionary behavior → Exercise participation	−0.056	0.021	2.772 *
Coronavirus anxiety → Stress → Precautionary behavior → Exercise participation	−0.019	0.010	2.000 *
Coronavirus anxiety → Stress → Precautionary behavior	−0.051	0.023	2.211 *
Stress → Precautionary behavior → Exercise participation	0.086	0.028	3.124 **

Note: * *p* < 0.05; ** *p* < 0.01.

## Data Availability

The data presented in this study are available from the corresponding author upon request.

## References

[1] World Health Organization Statement on the Second Meeting of the International Health Regulations (2005) Emergency Committee Regarding the Outbreak of Novel Coronavirus (2019-nCoV). https://www.who.int/news-room/detail/30-01-2020-statement-on-the-second-meeting-of-theinternational-health-regulations-(2005)-emergency-committee-regarding-theoutbreak-of-novel-coronavirus-(2019-ncov).

[2] Rosenbaum L. (2020). Facing COVID-19 in Italy—Ethics, Logistics, and Therapeutics on the Epidemic’s Front Line. N. Engl. J. Med..

[3] World Health Organization Impact of COVID-19 on People’s Livelihoods, Their Health and Our Food Systems. https://www.who.int/news/item/13-10-2020-impact-of-covid-19-on-people's-livelihoods-their-health-and-our-food-systems.

[4] Da Silva M.A., Singh-Manoux A., Brunner E.J., Kaffashian S., Shipley M.J., Kivimäki M., Nabi H. (2012). Bidirectional association between physical activity and symptoms of anxiety and depression: The Whitehall II study. Eur. J. Epidemiol..

[5] Kaur H., Singh T., Arya Y.K., Mittal S. (2020). Physical fitness and exercise during the COVID-19 pandemic: A qualitative enquiry. Front. Psychol..

[6] Shariat A., Cleland J.A., Hakakzadeh A. (2020). Home-based exercises during the COVID-19 quarantine situation for office workers: A commentary. Work.

[7] Lazarus R.S. (1991). Cognition and motivation in emotion. Am. Psychol..

[8] Lazarus R.S., Scherer K.R., Schorr A., Johnstone T. (2001). Relational meaning and discrete emotions. Appraisal Processes in Emotion.

[9] Rather R.A. (2021). Demystifying the effects of perceived risk and fear on customer engagement, co-creation and revisit intention during COVID-19: A protection motivation theory approach. J. Destin. Mark. Manag..

[10] Rather R.A. (2021). Monitoring the impacts of tourism-based social media, risk perception and fear on tourist’s attitude and revisiting behaviour in the wake of COVID-19 pandemic. Curr. Issues Tour..

[11] Taylor S. (2019). The Psychology of Pandemics: Preparing for the Next Global Outbreak of Infectious Disease.

[12] Asmundson G.J.G., Taylor S. (2020). Coronaphobia: Fear and the 2019-nCoV outbreak. J. Anxiety Disord..

[13] Asmundson G.J.G., Taylor S. (2020). How health anxiety influences responses to viral outbreaks like COVID-19: What all decision-makers, health authorities, and health care professionals need to know. J. Anxiety Disord..

[14] Broman-Fulks J.J., Berman M.E., Rabian B.A., Webster M.J. (2004). Effects of aerobic exercise on anxiety sensitivity. Behav. Res. Ther..

[15] Long B.C., Stavel R.V. (1995). Effects of exercise training on anxiety: A meta-analysis. J. Appl. Sport Psychol..

[16] Mackay G.J., Neill J.T. (2010). The effect of “green exercise” on state anxiety and the role of exercise duration, intensity, and greenness: A quasi-experimental study. Psychol. Sport Exerc..

[17] Çifçi F., Demir A. (2020). The Effect of Home-Based Exercise on Anxiety and Mental Well-Being Levels of Teachers and Pre-Service Teachers in COVID-19 Pandemic. Afr. Educ. Res. J..

[18] Hu S., Tucker L., Wu C., Yang L. (2020). Beneficial effects of exercise on depression and anxiety during the COVID-19 pandemic: A narrative review. Front. Psychiatry.

[19] López-Bueno R., Calatayud J., Ezzatvar Y., Casajús J.A., Smith L., Andersen L.L., López-Sánchez G.F. (2020). Association between current physical activity and current perceived anxiety and mood in the initial phase of COVID-19 confinement. Front. Psychiatry.

[20] Arnold M.B. (1960). Emotion and Personality.

[21] Moors A. (2014). Flavors of Appraisal Theories of Emotion. Emot. Rev..

[22] Moroń M., Biolik-Moroń M. (2021). Trait emotional intelligence and emotional experiences during the COVID-19 pandemic outbreak in Poland: A daily diary study. Personal. Individ. Differ..

[23] Pérez-Fuentes M.d.C., Molero Jurado M.d.M., Martos Martínez Á., Gázquez Linares J.J. (2020). Threat of COVID-19 and emotional state during quarantine: Positive and negative affect as mediators in a cross-sectional study of the Spanish population. PLoS ONE.

[24] Levkovich I., Shinan-Altman S. (2020). Impact of the COVID-19 pandemic on stress and emotional reactions in Israel: A mixed-methods study. Int. Health.

[25] Canet-Juric L., Andrés M.L., del Valle M., López-Morales H., Poó F., Galli J.I., Yerro M., Urquijo S. (2020). A Longitudinal Study on the Emotional Impact Cause by the COVID-19 Pandemic Quarantine on General Population. Front. Psychol..

[26] Adwas A., Jbireal J., Azab A. (2019). Anxiety: Insights into Signs, Symptoms, Etiology, Pathophysiology, and Treatment. S. Afr. J. Med. Sci..

[27] Higgins-Chen A.T., Abdallah S.B., Dwyer J.B., Kaye A.P., Angarita G.A., Bloch M.H. (2019). Severe Illness Anxiety Treated by Integrating Inpatient Psychotherapy With Medical Care and Minimizing Reassurance. Front. Psychiatry.

[28] Montgomery S.M., Cook D.G., Bartley M.J., Wadsworth M.E. (1999). Unemployment pre-dates symptoms of depression and anxiety resulting in medical consultation in young men. Int. J. Epidemiol..

[29] Salari N., Hosseinian-Far A., Jalali R., Vaisi-Raygani A., Rasoulpoor S., Mohammadi M., Rasoulpoor S., Khaledi-Paveh B. (2020). Prevalence of stress, anxiety, depression among the general population during the COVID-19 pandemic: A systematic review and meta-analysis. Glob. Health.

[30] Bhargava D., Trivedi H. (2018). A study of causes of stress and stress management among youth. Inst. Res. Adv..

[31] Chrousos G.P. (2009). Stress and disorders of the stress system. Nat. Rev. Endocrinol..

[32] Daviu N., Bruchas M.R., Moghaddam B., Sandi C., Beyeler A. (2019). Neurobiological links between stress and anxiety. Neurobiol. Stress.

[33] Dryhurst S., Schneider C.R., Kerr J., Freeman A.L.J., Recchia G., van der Bles A.M., Spiegelhalter D., van der Linden S. (2020). Risk perceptions of COVID-19 around the world. J. Risk Res..

[34] Anderson R.M., Heesterbeek H., Klinkenberg D., Hollingsworth T.D. (2020). How will country-based mitigation measures influence the course of the COVID-19 epidemic?. Lancet.

[35] Seale H., Dyer C.E.F., Abdi I., Rahman K.M., Sun Y., Qureshi M.O., Dowell-Day A., Sward J., Islam M.S. (2020). Improving the impact of non-pharmaceutical interventions during COVID-19: Examining the factors that influence engagement and the impact on individuals. BMC Infect. Dis..

[36] Leung G.M., Lam T.-H., Ho L.-M., Ho S.-Y., Chan B.H.Y., Wong I.O.L., Hedley A.J. (2003). The impact of community psychological responses on outbreak control for severe acute respiratory syndrome in Hong Kong. J. Epidemiol. Commun. Health.

[37] Griffin R.J., Dunwoody S., Zabala F. (1998). Public Reliance on Risk Communication Channels in the Wake of a Cryptosporidium Outbreak. Risk Anal..

[38] Novotny J.S., Gonzalez Rivas J.P., Kunzova S., Skladana M., Pospisilova A., Polcrova A., Medina Inojosa J.R., Lopez-Jimenez F., Geda Y.E., Stokin G.B. (2020). Association between stress and depressive symptoms and the COVID-19 pandemic. medRxiv.

[39] Charoensukmongkol P., Phungsoonthorn T. (2020). The effectiveness of supervisor support in lessening perceived uncertainties and emotional exhaustion of university employees during the COVID-19 crisis: The constraining role of organizational intransigence. J. Gen. Psychol..

[40] Chekroud S.R., Gueorguieva R., Zheutlin A.B., Paulus M., Krumholz H.M., Krystal J.H., Chekroud A.M. (2018). Association between physical exercise and mental health in 1·2 million individuals in the USA between 2011 and 2015: A cross-sectional study. Lancet Psychiatry.

[41] Deslandes A., Moraes H., Ferreira C., Veiga H., Silveira H., Mouta R., Pompeu F.A.M.S., Coutinho E.S.F., Laks J. (2009). Exercise and Mental Health: Many Reasons to Move. Neuropsychobiology.

[42] World Health Organization Coronavirus Diseases (COVID-19) Pandemic. https://www.who.int/emergencies/diseases/novel-coronavirus-2019.

[43] Ministry of Health Circuit Breaker to Minimize Further Spread of COVID-19. https://www.moh.gov.sg/news-highlights/details/circuit-breaker-to-minimise-further-spread-of-COVID-19.

[44] Goodwin R.D. (2003). Association between physical activity and mental disorders among adults in the United States. Prev. Med..

[45] Radloff L.S. (1977). The CES-D Scale: A Self-Report Depression Scale for Research in the General Population. Appl. Psychol. Meas..

[46] Zillmann D., Bryant J., Zillmann D., Bryant J. (1985). Affect, mood, and emotion as determinants of selective exposure. Selective Exposure to Communication.

[47] Heslop P., Smith G.D., Carroll D., Macleod J., Hyland F., Hart C. (2001). Perceived stress and coronary heart disease risk factors: The contribution of socio-economic position. Br. J. Health Psychol..

[48] Lutz R.S., Lochbaum M.R., Lanning B., Stinson L.G., Brewer R. (2007). Cross-lagged relationships among leisure-time exercise and perceived stress in blue-collar workers. J. Sport Exerc. Psychol..

[49] Hunter P.R., Colon-Gonzalez F., Brainard J.S., Rushton S. (2020). Impact of non-pharmaceutical interventions against COVID-19 in Europe: A quasi-experimental study. medRxiv.

[50] Lippi G., Henry B.M., Bovo C., Sanchis-Gomar F. (2020). Health risks and potential remedies during prolonged lockdowns for coronavirus disease 2019 (COVID-19). Diagnosis.

[51] Singapore Government What Are the Heightened Safe Distancing Measures for Workplaces during the Circuit Breaker Period?. https://www.gov.sg/article/what-are-the-heightened-safe-distancing-measures-for-workplaces-during-the-circuit-breaker-period.

[52] Lee S.A. (2020). Coronavirus Anxiety Scale: A brief mental health screener for COVID-19 related anxiety. Death Stud..

[53] Cohen S., Kamarck T., Mermelstein R. (1983). A global measure of perceived stress. J. Health Soc. Behav..

[54] Lee C.-K., Song H.-J., Bendle L.J., Kim M.-J., Han H. (2012). The impact of non-pharmaceutical interventions for 2009 H1N1 influenza on travel intentions: A model of goal-directed behavior. Tour. Manag..

[55] Godin G., Shephard R. (1985). A simple method to assess exercise behavior in the community. Can. J. Appl. Sport Sci..

[56] Tabachnick B.G., Fridell L.S. (2013). Using Multivariate Statistics.

[57] Podsakoff P.M., MacKenzie S.B., Lee J.-Y., Podsakoff N.P. (2003). Common method biases in behavioral research: A critical review of the literature and recommended remedies. J. Appl. Psychol..

[58] Kock N., Latan H., Noonan R. (2017). Common method bias: A full collinearity assessment method for PLS-SEM. Partial Least Squares Path Modeling: Basic Concepts, Methodological Issues and Applications.

[59] Ringle C.M., Wende S., Becker J.M. (2015). SmartPLS 3.

[60] Hair J.F., Hult G.T.M., Ringle C., Sarstedt M. (2022). A Primer on Partial Least Squares Structural Equation Modeling (PLS-SEM).

[61] Henseler J., Ringle C.M., Sarstedt M. (2015). A new criterion for assessing discriminant validity in variance-based structural equation modeling. J. Acad. Mark. Sci..

[62] Nitzl C., Roldan Jose L., Cepeda G. (2016). Mediation analysis in partial least squares path modeling: Helping researchers discuss more sophisticated models. Ind. Manag. Data Syst..

[63] Husky M.M., Kovess-Masfety V., Swendsen J.D. (2020). Stress and anxiety among university students in France during COVID-19 mandatory confinement. Compr. Psychiatry.

[64] Charles S.T., Piazza J.R., Mogle J., Sliwinski M.J., Almeida D.M. (2013). The wear and tear of daily stressors on mental health. Psychol. Sci..

[65] Galea S., Merchant R.M., Lurie N. (2020). The Mental Health Consequences of COVID-19 and Physical Distancing: The Need for Prevention and Early Intervention. JAMA Intern. Med..

[66] Swami V., Horne G., Furnham A. (2021). COVID-19-related stress and anxiety are associated with negative body image in adults from the United Kingdom. Personal. Individ. Differ..

[67] Reger M.A., Stanley I.H., Joiner T.E. (2020). Suicide Mortality and Coronavirus Disease 2019—A Perfect Storm?. JAMA Psychiatry.

[68] Shevlin M., McBride O., Murphy J., Miller J.G., Hartman T.K., Levita L., Mason L., Martinez A.P., McKay R., Stocks T.V.A. (2020). Anxiety, depression, traumatic stress and COVID-19-related anxiety in the UK general population during the COVID-19 pandemic. BJPsych Open.

[69] Tull M.T., Edmonds K.A., Scamaldo K.M., Richmond J.R., Rose J.P., Gratz K.L. (2020). Psychological Outcomes Associated with Stay-at-Home Orders and the Perceived Impact of COVID-19 on Daily Life. Psychiatry Res..

[70] Cypryańska M., Nezlek J.B. (2020). Anxiety as a mediator of relationships between perceptions of the threat of COVID-19 and coping behaviors during the onset of the pandemic in Poland. PLoS ONE.

[71] Raude J., Setbon M. (2009). Lay perceptions of the pandemic influenza threat. Eur. J. Epidemiol..

[72] Oshitani H. (2006). Potential benefits and limitations of various strategies to mitigate the impact of an influenza pandemic. J. Infect. Chemother..

[73] Singapore Sport Advisory on Tightened Measures for Sport and Physical Exercise during the Extended Circuit Breaker Period. https://www.sportsingapore.gov.sg/newsroom/media-releases/2020/advisory-on-tightened-measures-on-sport-and-physical-exercise.

[74] Ferguson N.M., Laydon D., Nedjati-Gilani G., Imai N., Ainslie K., Baguelin M., Bhatia S., Boonyasiri A., Cucunubá Z., Cuomo-Dannenburg G. (2020). Impact of Non-Pharmaceutical Interventions (NPIs) to Reduce COVID-19 Mortality and Healthcare Demand.

[75] Bavel J.J.V., Baicker K., Boggio P.S., Capraro V., Cichocka A., Cikara M., Crockett M.J., Crum A.J., Douglas K.M., Druckman J.N. (2020). Using social and behavioural science to support COVID-19 pandemic response. Nat. Hum. Behav..

[76] Li S., Stampfer M.J., Williams D.R., VanderWeele T.J. (2016). Association of religious service attendance with mortality among women. JAMA Intern. Med..

[77] Chan R.K. (2021). Tackling COVID-19 risk in Hong Kong: Examining distrust, compliance and risk management. Curr. Sociol..

[78] Belli S., Alonso C. (2021). COVID-19 pandemic and emotional contagion. Digithum.

[79] Chiu W., Oh G.E., Cho H. (2022). Impact of COVID-19 on consumers’ impulse buying behavior of fitness products: A moderated mediation model. J. Consum. Behav..

